# Supplementary material for: Low thermal conductivity of iron-silicon alloys at Earth’s core conditions with implications for the geodynamo
Source: Nat Commun. 2020 Jul 3;11:3332. doi: 10.1038/s41467-020-17106-7 (PMC7335046; doi:10.1038/s41467-020-17106-7)
Supplement: Supplementary file 1 — Supplementary Information [file 41467_2020_17106_MOESM1_ESM.pdf]

Supplementary Information for

Low thermal conductivity of iron-silicon alloys at Earth's core conditions with implications for the geodynamo

by Hsieh et al.

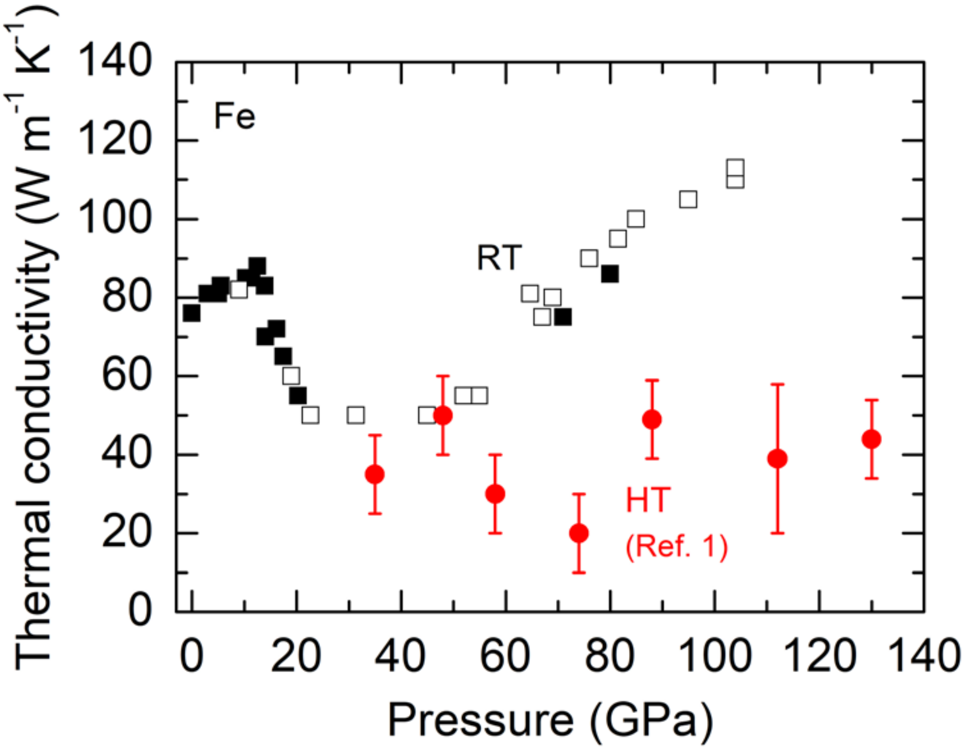

Supplementary Figure 1. Pressure dependence of the thermal conductivity of pure Fe at room temperature (black symbols) and high temperatures (red symbols). The measurement uncertainties at room temperature are ≈10% before 30 GPa, ≈20% at 60 GPa, and ≈25% at 120 GPa. The high temperature data were taken from Supplementary Ref. [1].

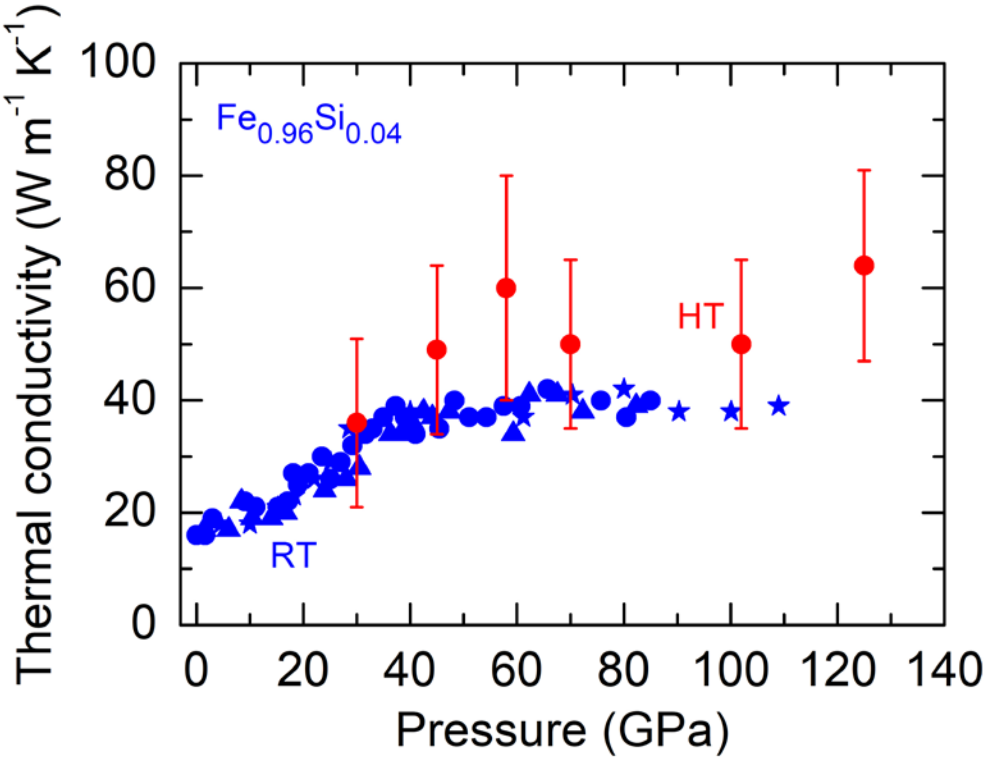

Supplementary Figure 2. Pressure dependence of the thermal conductivity of $Fe_{0.96}Si_{0.04}$ alloy at room temperature (blue symbols) and high temperatures (red symbols). The measurement uncertainties at room temperature are ≈10% before 30 GPa, ≈20% at 60 GPa, and ≈25% at 120 GPa. The temperature conditions of high temperature measurements are given in Supplementary Table 1.

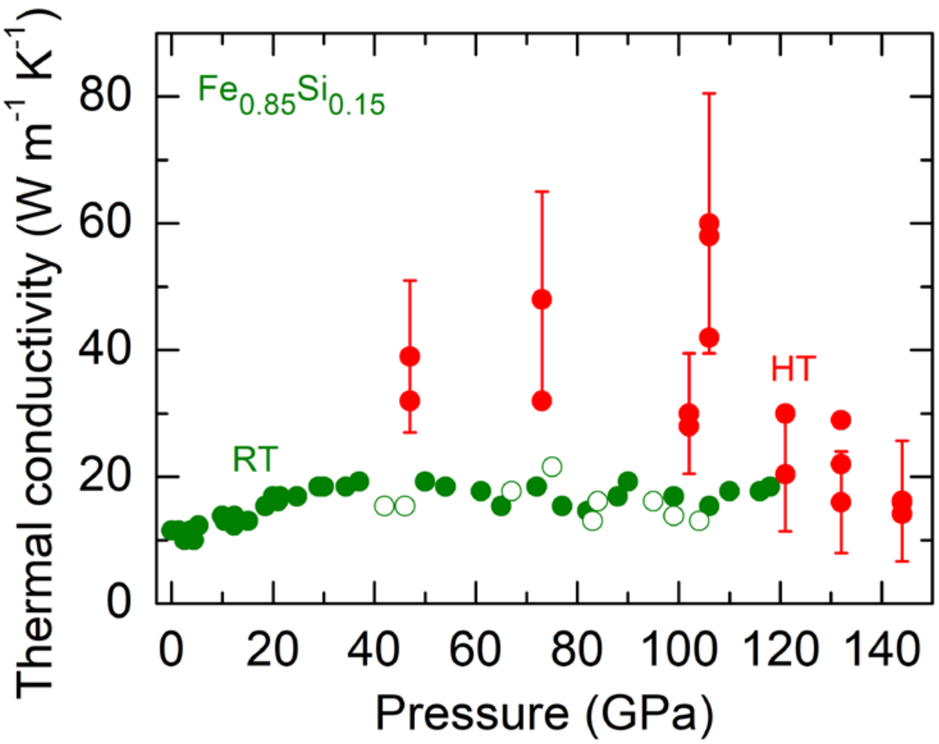

Supplementary Figure 3. Pressure dependence of the thermal conductivity of $Fe_{0.85}Si_{0.15}$ alloy at room temperature (green symbols) and high temperatures (red symbols). The measurement uncertainties at room temperature are ≈10% before 30 GPa, ≈20% at 60 GPa, and ≈25% at 120 GPa. The temperature conditions of high temperature measurements are given in Supplementary Table 3.

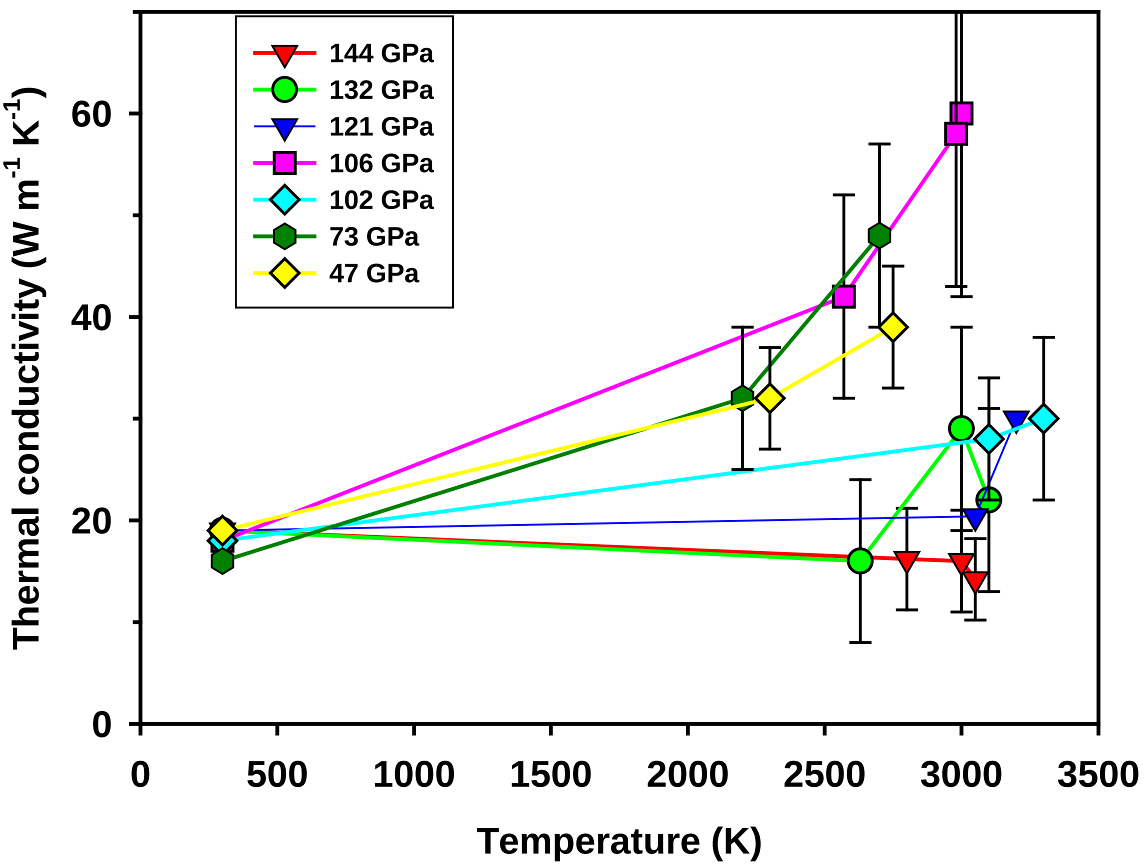

Supplementary Figure 4. Temperature dependence of the thermal conductivity of $Fe_{0.85}Si_{0.15}$ alloy determined in this work at various pressures.

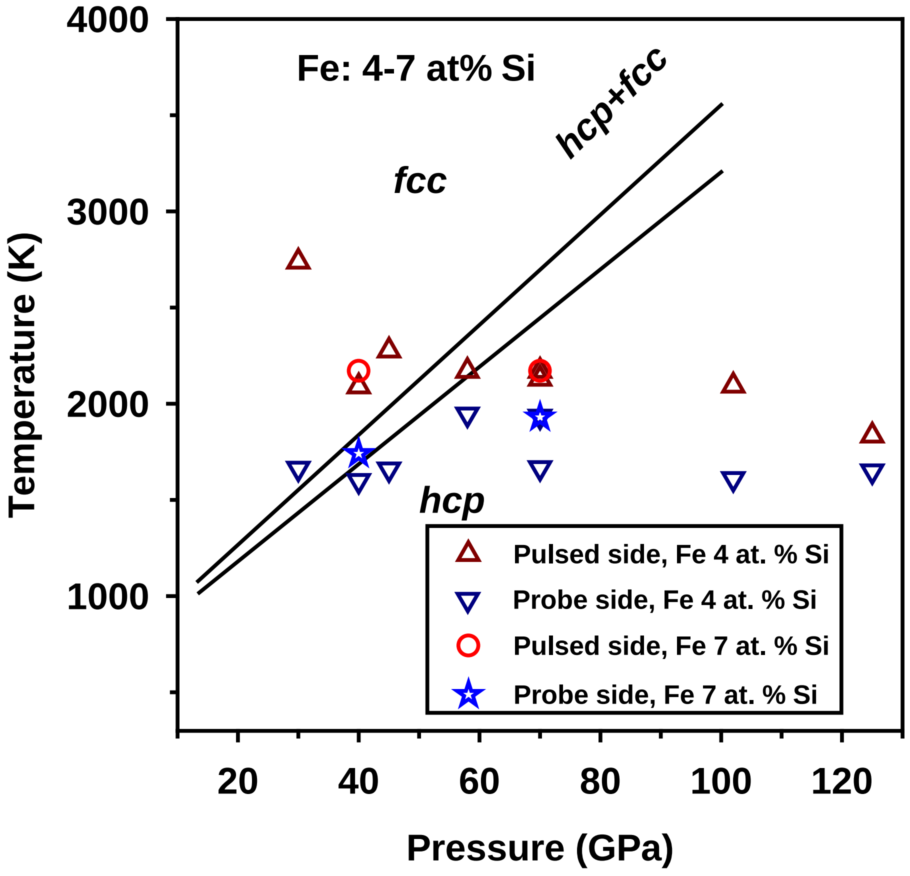

Supplementary Figure 5. *P*-*T* diagram illustrating the phase stability of $Fe_{0.93}Si_{0.07}$ alloy and conditions for our TH experiments. The phase diagram is taken from Komabayashi *et al.* (Supplementary Ref. [2]). Symbols are the *P*-*T* conditions of our high-temperature TH experiments for $Fe_{0.96}Si_{0.04}$ and $Fe_{0.93}Si_{0.07}$, where the symbol at the top (bottom) of each pressure represents measurement from the pulsed (probe) side of the sample. For instance, the dark brown up-triangles represent the *P*-*T* conditions of $Fe_{0.96}Si_{0.04}$, where the temperatures were measured from the pulsed side of the sample, while the blue stars represent the *P*-*T* conditions of $Fe_{0.93}Si_{0.07}$, where the temperatures were measured from the probe side of the sample. Solid lines are phase boundaries for $Fe_{0.93}Si_{0.07}$. Note that from Supplementary Ref. [2], the *P*-*T* diagram and phase boundary between *fcc*-*hcp* phases for $Fe_{0.96}Si_{0.04}$ is expected to be similar to the $Fe_{0.93}Si_{0.07}$.

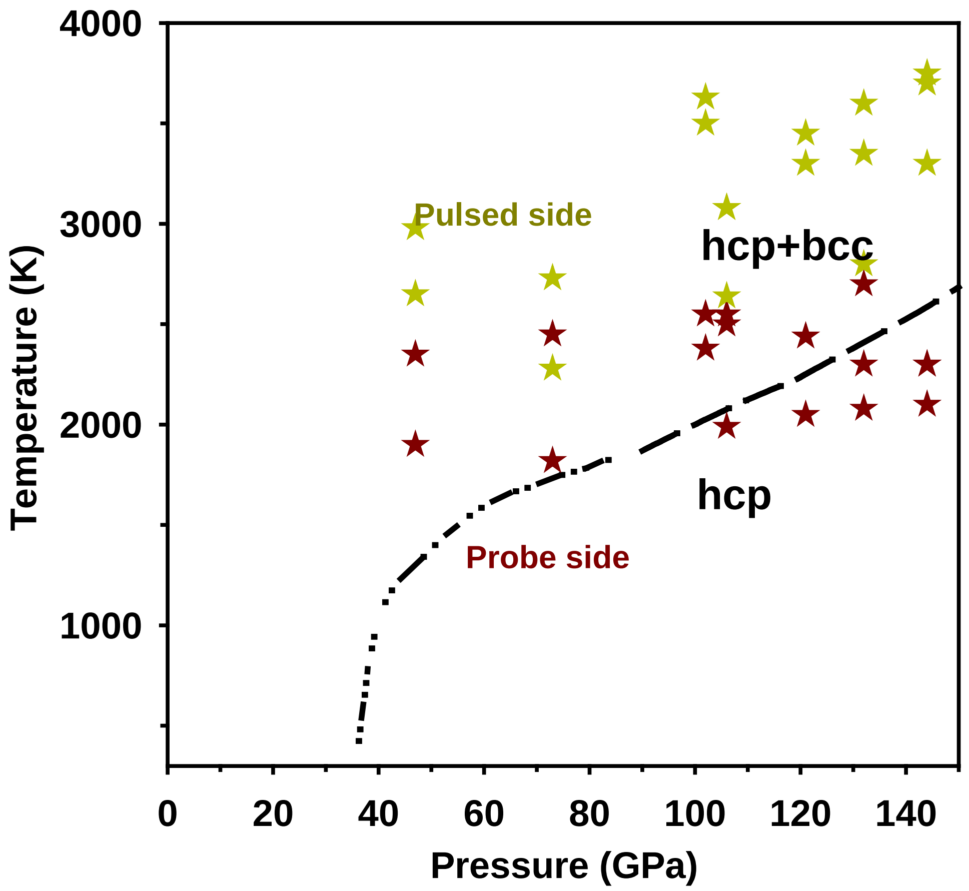

Supplementary Figure 6. *P*-*T* phase diagram of the $Fe_{0.85}Si_{0.15}$ alloy taken from Lin *et al.* (Supplementary Ref. [3]) illustrating the stability fields of various phases. Dark yellow (brown) stars are the *P*-*T* conditions of our high-temperature TH experiments collected from the pulsed (probe) side of the sample, and represent the range of sample temperature variation measured by radiative temperature measurements from pulsed (probe) side of the sample.

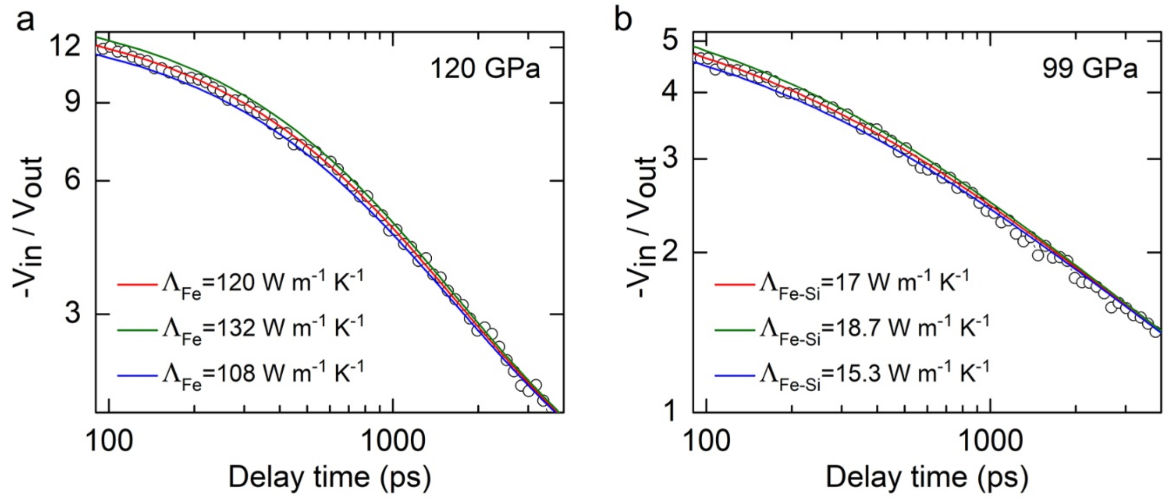

Supplementary Figure 7. Comparison of representative TDTR data (open circles) with thermal model calculations (solid curves) for **a** Fe at 120 GPa and **b** $Fe_{0.85}Si_{0.15}$ alloy at 99 GPa. Different solid curves are calculations using different input thermal conductivity Λ of Fe and $Fe_{0.85}Si_{0.15}$ alloy. When Fe is compressed to 120 GPa and $Fe_{0.85}Si_{0.15}$ alloy to 99 GPa, Λ_{Fe}=120 and $\Lambda_{Fe\text{-}Si}$=17 W m^{-1} K^{-1} (red curves), respectively, offer a best-fit to the data (see Supplementary Table 4 for other input parameters for Fe at 120 GPa). The ratio $-V_{in}/V_{out}$ is most sensitive to the Λ of samples during delay times of few hundred ps, particularly from 100 to 500 ps[4,5]. A 10% variation in Λ (green and blue curves) shows a clear deviation from the best-fit to the data, indicating the thermal model fitting and derived Λ_{Fe} and $\Lambda_{Fe\text{-}Si}$ are precise and reliable due to the high quality data and sample geometry.

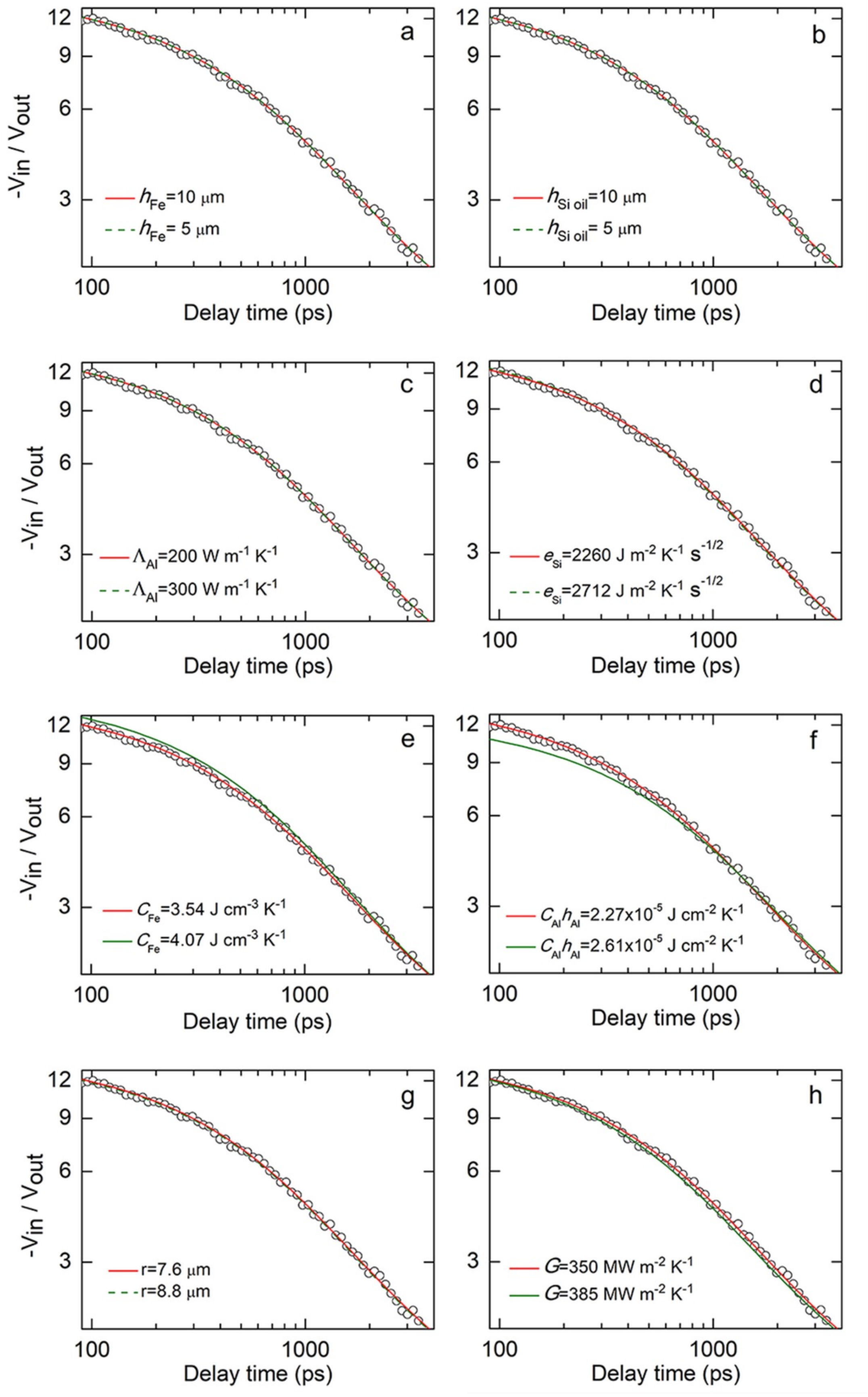

Supplementary Figure 8. Tests of sensitivity of the thermal model to input parameters for Fe at 120 GPa in TDTR measurements. Here we fix the Fe thermal conductivity Λ_{Fe} to be 120 W

m^{-1} K^{-1}, as derived in Supplementary Fig. **7a**, using input parameters listed in Supplementary Table 4. **a** and **b** If variations in the thicknesses of Fe (h_{Fe}) and silicone oil ($h_{Si\ oil}$), respectively, were as large as 50%, the model calculations show identical fits to the data, which indicates that uncertainties in the h_{Fe} and $h_{Si\ oil}$ have essentially no effect on the derived Λ_{Fe}. **c** The large Al thermal conductivity, Λ_{Al}, has very minor effect on the Λ_{Fe}. **d** An example variation in the thermal effusivity of the pressure medium silicone oil, $e=(\Lambda_{Si}C_{Si})^{1/2}$, by 20% still shows nearly the same model calculation, i.e., its uncertainty does not influence the derived Λ_{Fe}. **e** An example uncertainty in the volumetric heat capacity of Fe, C_{Fe}, by 15% (3.54 to 4.07 J cm^{-3} K^{-1}) only slightly deviates the model calculation from the data, which requires Λ_{Fe} to decrease slightly to 108 W m^{-1} K^{-1} to re-fit the data, i.e., propagating approximately 10% uncertainty to the derived Λ_{Fe}. **f** The major measurement uncertainty is from the uncertainty in Al heat capacity per unit area, product of volumetric heat capacity and thickness, $C_{Al}\,h_{Al}$, as the ratio $-V_{in}/V_{out}$ at few hundred ps delay time scales inversely with the $C_{Al}\,h_{Al}$ [4]. For instance, a 15% uncertainty requires approximately 20% change in the Λ_{Fe} to re-fit the data. **g** Laser spot size changed by as large as 15% (7.6 to 8.8 μm) still shows the same model calculation, and thus does not affect the Λ_{Fe}. **h** Variations in the thermal conductance of Al/Fe interface and Al/silicone oil interface, G, only slightly influence the Λ_{Fe}. Variations in G mostly change the slope of model calculation at delay times longer than 1000 ps [4,5]. An example of 10% uncertainty has already made the model calculation deviating from the data, in particular after 1000 ps. The uncertainty in G is typically less than 10%, which only induces 4% uncertainty in the derived Λ_{Fe}.

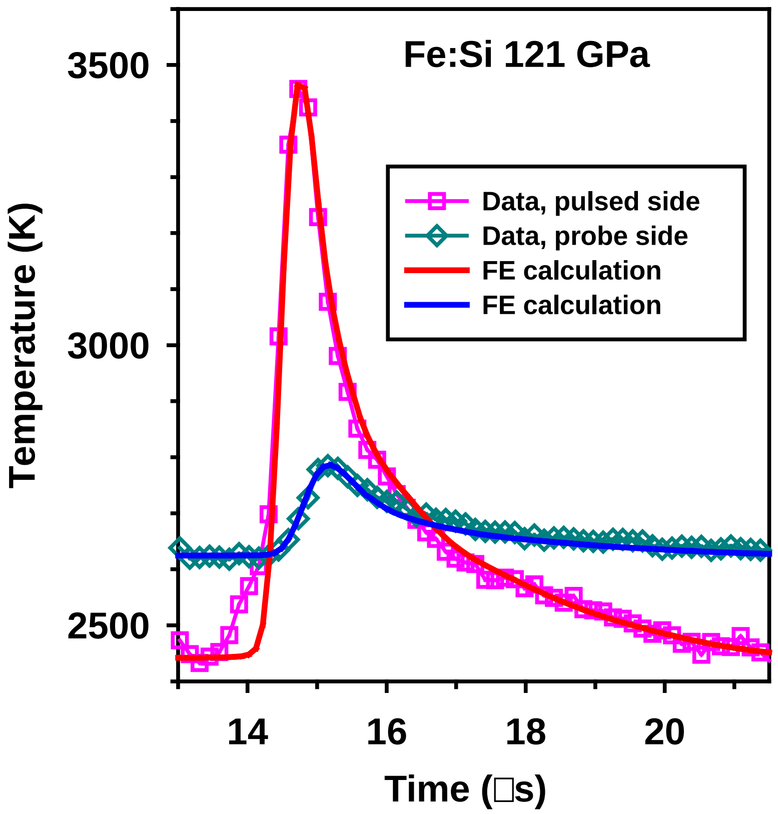

Supplementary Figure 9. Temperature of $Fe_{0.85}Si_{0.15}$ foils at 121 GPa during flash heating at high initial temperature. Data symbols: spectroradiometry measurements, lines: the best manual fit to the data using finite-element (FE) model calculations yielding thermal conductivity of $Fe_{0.85}Si_{0.15}$. The departure of the measured and calculated curves for the pulsed side near the time of the pulse arrival (14 μs) is an instrumental artifact due to a limited time resolution of the system.

Supplementary Table 1. High pressure-temperature thermal conductivity data for $Fe_{0.96}Si_{0.04}$

P (GPa)	T_{ave} (K)	$\Lambda_{Fe\text{-}Si}$ (W m^{-1} K^{-1})	P (GPa)	T_{ave} (K)	$\Lambda_{Fe\text{-}Si}$ (W m^{-1} K^{-1})
30	2050	36	45	2050	49
58	2050	60	70	2050	50
102	2050	50	125	2050	64

T_{ave}: Average measurement temperature; $\Lambda_{Fe\text{-}Si}$: Thermal conductivity of $Fe_{0.96}Si_{0.04}$

Supplementary Table 2. High pressure-temperature thermal conductivity data for $Fe_{0.93}Si_{0.07}$

P (GPa)	T_{ave} (K)	Λ_{Fe-Si} (W m^{-1} K^{-1})	P (GPa)	T_{ave} (K)	Λ_{Fe-Si} (W m^{-1} K^{-1})
40	2050	55	70	2050	49

T_{ave}: Average measurement temperature; Λ_{Fe-Si}: Thermal conductivity of $Fe_{0.93}Si_{0.07}$

Supplementary Table 3. High pressure-temperature thermal conductivity data for $Fe_{0.85}Si_{0.15}$

P (GPa)	T_{ave} (K)	$\Lambda_{Fe\text{-}Si}$ (W m^{-1} K^{-1})	P (GPa)	T_{ave} (K)	$\Lambda_{Fe\text{-}Si}$ (W m^{-1} K^{-1})
47	2750	39	73	2700	48
	2300	32		2200	32
102	3100	28	106	3000	60
	3300	30		2980	58
				2570	42
121	3050	20.4	132	2630	16
	3200	30		3000	29
				3100	22
144	3050	14.2			
	3000	16			
	2800	16.2			

T_{ave}: Average measurement temperature; $\Lambda_{Fe\text{-}Si}$: Thermal conductivity of $Fe_{0.85}Si_{0.15}$

Supplementary Table 4. Input parameters in the thermal model for Fe at 120 GPa and 300 K in TDTR measurements

P (GPa)	C_{Fe} (J cm^{-3} K^{-1})	C_{Al} (J cm^{-3} K^{-1})	h_{Al} (nm)*	$e=(\Lambda_{Si}C_{Si})^{1/2}$ (J m^{-2} K^{-1} s$^{-1/2}$)	r (μm)	$h_{Fe/Si\ oil}$ (μm)	Λ_{Al} (W m^{-1} K^{-1})	G (MW m^{-2} K^{-1})
120	3.54	2.684	84.8	2260	7.6	10	200	350

*In this experimental run, the Al thickness at ambient pressure is 100.3 nm.
C_{Fe}: Fe heat capacity, C_{Al}: Al heat capacity, h_{Al}: Al thickness, e: silicone oil thermal effusivity, r: laser spot size, h_{Fe}: Fe thickness, $h_{Si\ oil}$: silicone oil thickness, Λ_{Al}: Al thermal conductivity, G: thermal conductance of Al/Fe and Al/silicone oil interfaces.

Supplementary Table 5. Input parameters in the thermal model for $Fe_{0.85}Si_{0.15}$ at 121 GPa and 2400–3500 K (T_{ave}=3050 K) in FE calculation (Supplementary Fig. 9).

P (GPa)	$C_{Fe:Si}$ (J kg^{-1} K^{-1})	C_{KCl} (J kg^{-1} K^{-1})	$\rho_{Fe:Si}$ (kg m^{-3})	ρ_{KCl} (kg m^{-3})	$h_{Fe:Si}$ (μm)	r_L (μm)	$\Lambda_{Fe:Si}$ (W m^{-1} K^{-1})	Λ_{KCl} (W m^{-1} K^{-1})
121	700	690	10500	5218	1.86	12.5	20.4	60

$C_{Fe:Si}$: $Fe_{0.85}Si_{0.15}$ specific heat capacity; C_{KCl}: KCl specific heat capacity; $\rho_{Fe:Si}$: $Fe_{0.85}Si_{0.15}$ density; ρ_{KCl}: KCl density; $h_{Fe:Si}$: $Fe_{0.85}Si_{0.15}$ thickness; r: laser spot size (FWHM); $\Lambda_{Fe:Si}$: $Fe_{0.85}Si_{0.15}$ thermal conductivity; Λ_{KCl}: KCl thermal conductivity.

Supplementary Note 1

Effects of pressure, temperature, and Si alloying on the thermal conductivity of Fe-Si alloys

Thermal conductivity of Fe or Fe-rich alloy is dominated by the electrical conductivity. The electrical conductivity (σ) and resistivity (inverse of conductivity) are strong functions of temperature (*T*) (1/σ is proportional to *T*). However, the temperature dependence of thermal conductivity should be weaker as it can be determined via the Wiedemann–Franz (WF) law $k = L \times \sigma \times T$, where *k* is the thermal conductivity and σ the electrical conductivity, and L the Lorenz number. That is why a small change in the *T* dependence of resistivity with pressure would result in a change of the *T* dependence of thermal conductivity, which can increase or decrease with *T* (Supplementary Fig. 4). This *T* dependence of thermal conductivity may also vary with the Si composition, making k decreasing with *T* (as in Supplementary Ref. [1]) or increasing with *T* (as in this work at 106 GPa, Supplementary Fig. 4).

Supplementary References

1. Konôpková, Z., McWilliams, R. S., Gómez-Pérez, N. &Goncharov, A. F. Direct measurement of thermal conductivity in solid iron at planetary core conditions. *Nature* **534**, 99–101 (2016).
2. Komabayashi, T. *et al.* Phase transition boundary between fcc and hcp structures in Fe-Si alloy and its implications for terrestrial planetary cores. *Am. Mineral.* **104**, 94–99 (2019).
3. Lin, J. F. *et al.* Phase relations of Fe-Si alloy in Earth's core. *Geophys. Res. Lett.* **36**, L06306 (2009).
4. Zheng, X., Cahill, D. G., Krasnochtchekov, P., Averback, R. S. &Zhao, J. C. High-throughput thermal conductivity measurements of nickel solid solutions and the applicability of the Wiedemann-Franz law. *Acta Mater.* **55**, 5177–5185 (2007).
5. Cahill, D. G. &Watanabe, F. Thermal conductivity of isotopically pure and Ge-doped Si epitaxial layers from 300 to 550 K. *Phys. Rev. B* **70**, 235322 (2004).
